# A Small-Animal Pharmacokinetic/Pharmacodynamic PET Study of Central Serotonin 1A Receptor Occupancy by a Potential Therapeutic Agent for Overactive Bladder

**DOI:** 10.1371/journal.pone.0075040

**Published:** 2013-09-23

**Authors:** Yosuke Nakatani, Michiyuki Suzuki, Masaki Tokunaga, Jun Maeda, Miyuki Sakai, Hiroki Ishihara, Takashi Yoshinaga, Osamu Takenaka, Ming-Rong Zhang, Tetsuya Suhara, Makoto Higuchi

**Affiliations:** 1 Tsukuba Research Laboratories, Eisai Co, Ltd., Tsukuba, Ibaraki, Japan; 2 Molecular Imaging Center, National Institute of Radiological Sciences, Chiba, Chiba, Japan; 3 Graduate School of Medicine, Tohoku University, Sendai, Miyagi, Japan; Tokyo Metropolitan Institute of Medical Science, Japan

## Abstract

Serotonin 1A (5-HT_1A_) receptors have been mechanistically implicated in micturition control, and there has been a need for an appropriate biomarker surrogating the potency of a provisional drug acting on this receptor system for developing a new therapeutic approach to overactive bladder (OAB). Here, we analyzed the occupancy of 5-HT_1A_ receptors in living Sprague-Dawley rat brains by a novel candidate drug for OAB, E2110, using positron emission tomography (PET) imaging, and assessed the utility of a receptor occupancy (RO) assay to establish a pharmacodynamic index translatable between animals and humans. The plasma concentrations inducing 50% RO (EC_50_) estimated by both direct and effect compartment models were in good agreement. Dose-dependent therapeutic effects of E2110 on dysregulated micturition in different rat models of pollakiuria were also consistently explained by achievement of 5-HT_1A_ RO by E2110 in a certain range (≥ 60%). Plasma drug concentrations inducing this RO range and EC_50_ would accordingly be objective indices in comparing pharmacokinetics-RO relationships between rats and humans. These findings support the utility of PET RO and plasma pharmacokinetic assays with the aid of adequate mathematical models in determining the *in vivo* characteristics of a drug acting on 5-HT_1A_ receptors and thereby counteracting OAB.

## Introduction

Overactive bladder (OAB) is a pathological condition symptomatically diagnosed based upon “a symptom developing urinary urgency with or without urge incontinence and usually developing frequent urination with no proven infection or other obvious pathological factors [1].”, and is one of the most common diseases in the elderly. Most OAB cases are idiopathic, and anticholinergic agents are frequently used for its treatment. However, it is difficult to maintain sufficient dosage of an anticholinergic agent for expected efficacy without causing significant adverse events, such as dry mouth, gastrointestinal disorder, and urinary retention, thereby limiting its use [2–4].

**Figure 1:**
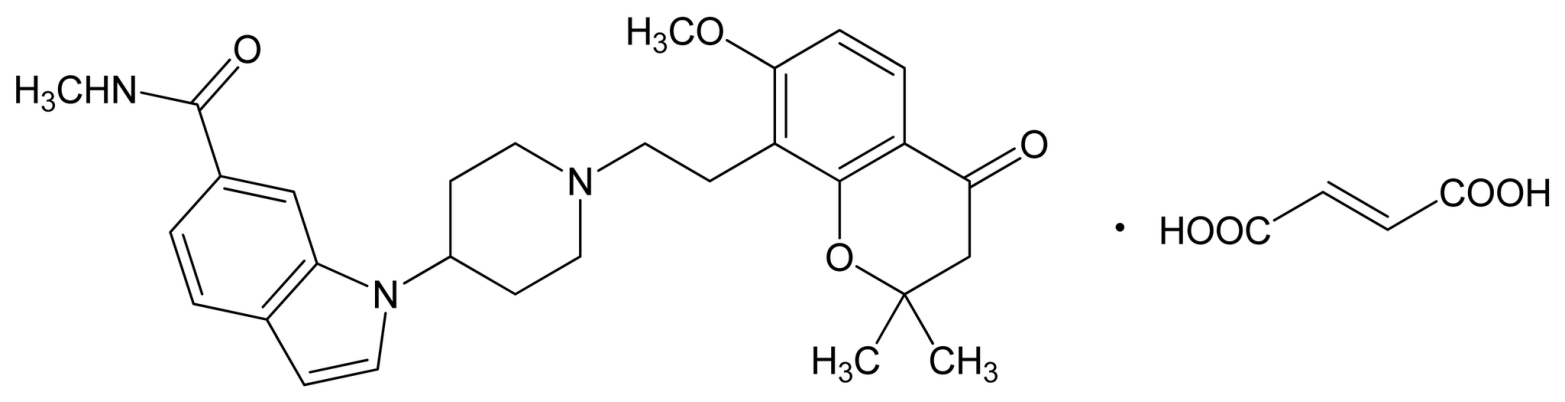
Chemical structure of E2110.

Several central nervous system (CNS) transmitter systems, including adrenaline, noradrenaline, gamma-aminobutyric acid, opioids, dopamine, and glutamate transmissions are known to be involved in micturition control. Serotonin (5-HT) and its receptors may also play an important role in the regulation of micturition reflex. Pharmacological studies suggested that serotonin 1A (5-HT_1A_) receptor stimulation by administration of its agonist, 8-hydroxy-2-(di-n-propylamino)-tetralin (8-OH-DPAT), decreased the volume threshold for bladder contractions and facilitated voiding. On the other hand, N-[2-[4-(2-methoxyphenyl)-1-piperazinyl] ethyl]-N-(2-pyridinyl)cyclohexanecarboxamide (WAY-100635), a silent 5-HT_1A_ receptor antagonist, increased residual bladder volume and thus markedly reduced voiding efficiency [5]. Compounds with higher selectivity for 5-HT_1A_ receptors may therefore provide a significant option for the treatment of OAB. 1-{1-[2-(7-Methoxy-2,2-dimethyl-4-oxochroman-8-yl) ethyl] piperidine-4-yl}-N-methyl-1H-indole-6-carboxamide fumarate (E2110; Figure 1) is a novel compound with high selectivity and affinity for 5-HT_1A_ receptors in the rodent brain *in vitro*. [^35^S] guanosine-5’-O-(3-thio)-triphosphate binding studies have demonstrated that E2110 is a full antagonist of 5-HT_1A_ receptors.

As the occupancy of target receptors by a therapeutic agent is intimately correlated with the intensity of its pharmacological effects, measurements of receptor occupancy (RO) may offer an objective index for assessing pharmacokinetics (PK) and pharmacodynamics (PD) of such a drug or drug candidate. Positron emission tomography (PET) has been recognized as an important tool in drug development, since it is capable of providing valuable information on PK, blood-brain barrier (BBB) penetration, and PD of a receptor ligand, as exemplified by RO, in living brains of diverse species including rodents and humans. Translation of target RO data from preclinical species to humans may be a viable strategy for predicting clinical efficacy and optimal dosage of a novel therapeutic agent, given that the relationship between RO and clinical effects has been established.

This approach relies upon an understanding of the relationship between drug concentrations in plasma and CNS that results in a specific level of target occupancy. A PK/PD modeling approach has also been used to describe the relationship between drug concentrations and RO. A maximum effect (E_max_) model to describe this relationship was typically applied to the PD assay, but such “static” approach could be of insufficient accuracy, particularly in case of distribution of the drug from plasma to brain or binding kinetics of the drug at the receptor being relatively slow. An optimal assessment requires determination of RO and plasma concentration at multiple time points postdose. Indirect or effect compartment models may be more appropriate than direct models to describe the relationship between occupancy and plasma concentration in treatment with a drug with delayed BBB penetration and/or target PD [6].

In the present study, we examined the utility of 5-HT_1A_ receptor PET imaging for obtaining a surrogate marker for assessing therapeutic effects of E2110 on urinary bladder dysfunctions. Occupancy of central 5-HT_1A_ receptors by E2110 was determined in rats using PET with a radiotracer, [^11^C] WAY-100635. A compartmental PK/PD modeling approach was then utilized to describe the relationship between plasma concentration of E2110 and 5-HT_1A_ RO by E2110 in several regions of rat brains at different time points after its oral administration. The results have supported the preclinical use of PET in combination with analytical models to predict PK/PD of a drug in humans.

## Materials and Methods

### Animals

#### 1): Ethics statement

The research protocols of the present work were approved by the Animal Ethics Committee of the National Institute of Radiological Sciences and the Animal Ethics Committee of Eisai Co. Ltd., and were performed in accordance with the Principles of Laboratory Animal Care (NIH publication No. 85-23, revised 1985).

#### 2): Materials and maintenance conditions

Male and female Sprague-Dawley (SD) rats were purchased from Japan SLC (Hamamatsu, Japan) and Charles River Japan Inc. (Kanagawa, Japan). All rats were kept in animal rooms maintained at 20-26°C and illuminated from 7 am to 7 pm daily, with *ad libitum* access to food and water.

### Reagents

E2110 was synthesized at Eisai Co., Ltd. (Japan). WAY-100635 maleate, 5-HT and 8-OH-DPAT were from Sigma-Aldrich (St. Louis, MO, USA). [^3^H] 2'-methoxyphenyl-(N-2'-pyridinyl)-p-fluoro-benzamidoethyipiperazine ([^3^H] MPPF) was from PerkinElmer Life & Analytical Sciences (Boston, MA, USA). Tamsulosin hydrochloride was purchased from WAKO Pure Chemical Industries (Osaka, Japan). All other reagents and chemicals were of analytical grade, and were commercially available.

### Binding assay in rat hippocampal membrane fraction

Rat hippocampal samples were weighed, homogenized in 10-fold volume of 50 mM Tris-HCl (pH 7.4) on an ice bath, and centrifuged (50,000×g, 4°C, 20 min). The precipitate was further homogenized in a 10-fold volume of 50 mM Tris-HCl (pH 7.4) on an ice bath and centrifuged (50,000×g, 4°C, 20 min). The obtained precipitate was homogenized in 50 mM Tris-HCl (pH 7.4) to render a concentration of 100 mg tissue eq./mL. The obtained microsomal fraction was stored as a receptor stock solution at -80°C until use. In saturation binding experiments, the suspension of hippocampal membranes (5 mg tissue eq./tube) in the tube was incubated with different concentrations (0.03–7.77 nM) of [^3^H] MPPF in a total 1-ml reaction volume at 25°C for 60 min. The reaction was terminated, and bound radioactivity was separated from free radioligands by filtering and washing three times with 3 ml of 50 mM Tris-HCl (pH 7.4). The radioactivity content of the filters in 5 ml of liquid scintillator (Atomlight; PerkinElmer) was counted by TR2500 scintillation counter (PerkinElmer Life & Analytical Sciences). The specific binding was determined as the difference between total and nonspecific binding, measured in the absence and presence of 100 µM 5-HT. Data were analyzed by Scatchard plot to estimate equilibrium constant (K_d_) values. For the competition experiments, binding assays were carried out using 0.3 nM [^3^H] MPPF in the presence of various concentrations of E2110 (0.01 to 10 nM) or WAY-100635 (0.03 to 30 nM). The IC_50_ values for E2110 and WAY-100635 were corrected to the K_i_ values using the K_d_ values of [^3^H] MPPF according to the following equation:

$$K_{i}=\frac{{IC}_{50}}{1+\frac{\left[radioligand\right]}{K_{d}}}$$ (1)

where [radioligand] means the concentration of the radioligand used.

### Protein binding of E2110 in rat plasma

The *in vitro* unbound fraction of E2110 in male SD rat plasma was quantified by equilibrium dialysis. One mL of the E2110 spiked sample (100, 1000 and 10000 ng/mL) and phosphate buffered saline (PBS) were applied to one and the other chambers of a dialysis cell, respectively. The cartridge was incubated in a water bath for 24 hr at 37°C. After incubation, aliquots were sampled from both chambers and submitted to determination of the concentration of E2110 in each matrix by liquid chromatography-tandem mass spectrometry. Unbound fractions for each compound were calculated as the ratio of E2110 concentration from the PBS side to that from the plasma side of the dialysis apparatus.

### Radioligand synthesis

[^11^C] WAY-100635 was prepared by ^11^C-acylation of WAY-100634 with [^11^C] cyclohexanecarbonyl chloride as previously described in detail [7]. Semi-preparative reverse-phase HPLC was used for the purification of [^11^C] WAY-100635. Radiochemical purity of the radioligand was more than 95%. Average specific radioactivity of [^11^C] WAY-100635 was 196.4 ± 83.4 GBq/mmol at the end of synthesis (EOS). All injections of [^11^C] WAY-100635 were given within 30 min after EOS.

### PET data acquisition

All PET scans for rats were performed with a small animal-dedicated microPET FOCUS 220 system (Siemens Medical Solutions USA, Knoxville, TN, USA), which yields a 25.8 cm (transaxial) × 7.6 cm (axial) field of view (FOV) and a spatial resolution of 1.3 mm full width at half maximum at the center of FOV [8]. The rats were anesthetized with 1.5-2% isoflurane in air (2 L/min flow rate). Their body temperature was controlled using homeothermic controller and plate with a rectal probe, and their heart rates and arterial oxygen saturations were continuously measured by a pulse oximeter (CANL-425SVA; Med Associates, St. Albans, VT, USA). We also monitored respiration rates of these animals with a custom-made monitoring system (Nagano Electronics, Hitachinaka, Japan). A 20-min transmission scan for attenuation correction was performed using a spiraling ^68^Ge-^68^Ga point source. Subsequently, list-mode scans were carried out for 90 min. All list-mode data were sorted and Fourier rebinned into two-dimensional sinograms (frames: 4 × 1, 8 × 2, and 14 × 5 min). Images were thereafter reconstructed using two-dimensional filtered back-projection with a 0.5-mm Hanning filter. [^11^C]-WAY-100635 was injected via the tail vein as a single bolus at the start of emission scanning. The injected dose of the radiotracer was 85.3 ± 28.6 MBq/rat (mean ± SD).

### PET data analysis

Anatomical regions of interest (ROIs) were placed on the medial prefrontal cortex (MPFC) and dorsal raphe nucleus (DRN) using PMOD^®^ image software (PMOD Group, Zurich, Switzerland) with reference to the MRI template. Radioligand binding was examined by calculating the binding potential (*BP*_ND_; ratio at equilibrium of specifically-bound radioligand to that of nondisplaceable radioligand in tissue) based on a simplified reference tissue model (SRTM) [9] using the cerebellar time-radioactivity curve as reference.

Occupancy of 5-HT_1A_ receptors by E2110 was calculated using the following equation:

$$RO(\%)=\frac{{BP}_{ND,baseline}-{BP}_{ND,drug}}{{BP}_{ND,baseline}}\times 100$$ (2)

where *BP*_ND, baseline_ and *BP*_ND_, _drug_ are estimated *BP*_ND_ at baseline and following drug administration, respectively.

### Rat RO study

To examine the dose-RO relationship at a single time point, four male SD rats, which had undergone a baseline PET imaging in advance, were scanned with PET at 4 hours after being pretreated with 4 oral doses (0.3, 1, 3, 10 mg/kg) of E2110. In this assay, approximately 250 µL of blood samples were collected from the tail vein upon initiation of the scan for quantification of plasma concentration of E2110. We further examined RO at different time points after the drug administration by conducting PET scans of four male SD rats at 2, 4, 6 or 8 hours after pretreatment with a single oral dose of 1 mg/kg E2110. In addition to this series of PET assays for male rats, pilot PET scans for female rats (n = 3) were carried out at baseline and 4 hours after oral administration of 0.1 mg/kg E2110 to examine whether relationships between plasma concentration of E2110 and RO by this drug in the brain are consistent between two genders. In all experiments, scans for the same individual receiving E2110 were conducted more than 1 week apart.

### PK study in rats

E2110 was administered orally to male and female SD rats, (4 animals/sex) at a dose of 1 mg/kg. Blood samples were collected from the jugular veins by heparinized syringes at 0.25, 0.5, 1, 2, 4, 6, and 8 hours after dosing.

### Quantitative bioanalysis

Concentrations of E2110 were determined in plasma or PBS by liquid chromatography - tandem mass spectrometry. Samples (5 µL) were injected into an L-column ODS (35 × 2.1 mm; 5 µM, Chemical Evaluation and Research Institute, Tokyo, Japan) with a flow rate of 0.6 mL/min. We used 0.1% formic acid in water and acetonitrile as mobile phase A and B, respectively. The gradient elution started with a gradient from 0% to 100% mobile phase B for 2 min, then 1 min of 100% B, followed by a gradient of 100% to 0% for 0.01 min at the end, at a flow rate of 0.6 ml/min. The mass spectrometer (Quattro Ultima-Pt; Micromass, Waters Ltd., Manchester, UK) was operated in a positive ion multiple reaction monitoring mode. E2110 was monitored using mass transitions of 490.4/459.2. Tamsulosin was used as an internal standard, and was monitored using a mass transition of 409.3/200.0. The lower limit of quantification of E2110 in plasma was 0.5 ng/mL.

### PK/PD modeling

#### 1) Analysis of dose-RO relationships

As described in previous studies on 5-HT_1A_ receptor antagonists [10–12] the relationship between RO and plasma drug concentration at equilibrium can be described according to the law of mass action by the curvilinear function, based on the assumption that the free brain concentration is equal to free plasma concentration and the free fraction in plasma is not concentration-dependent:

$$RO(\%)=\frac{{RO}_{\max}}{K_{d}^{app}+C_{p}}C_{p}$$ (3)

where RO_max_ is the maximum occupancy that can be induced by E2110, K_d_^app^ the apparent equilibrium constant, and C_p_ the plasma concentration of E2110. This model is analogous to the E_max_ model:

$$E=\frac{E_{\max}}{{EC}_{50}+C_{p}}C_{p}$$ (4)

where E_max_ (RO_max_) is the maximum effect (maximum RO) and EC_50_ (= K_d_) is the E2110 plasma concentration required to produce 50% of E_max_. EC_50_ can be converted to “free” based EC_50_ by multiplying the unbound fraction ratio in plasma. In this study, the relationship between E2110 plasma concentration and RO was characterized by fitting the E_max_ model to the experimental data.

#### 2) Time-course study

**Figure 2:**
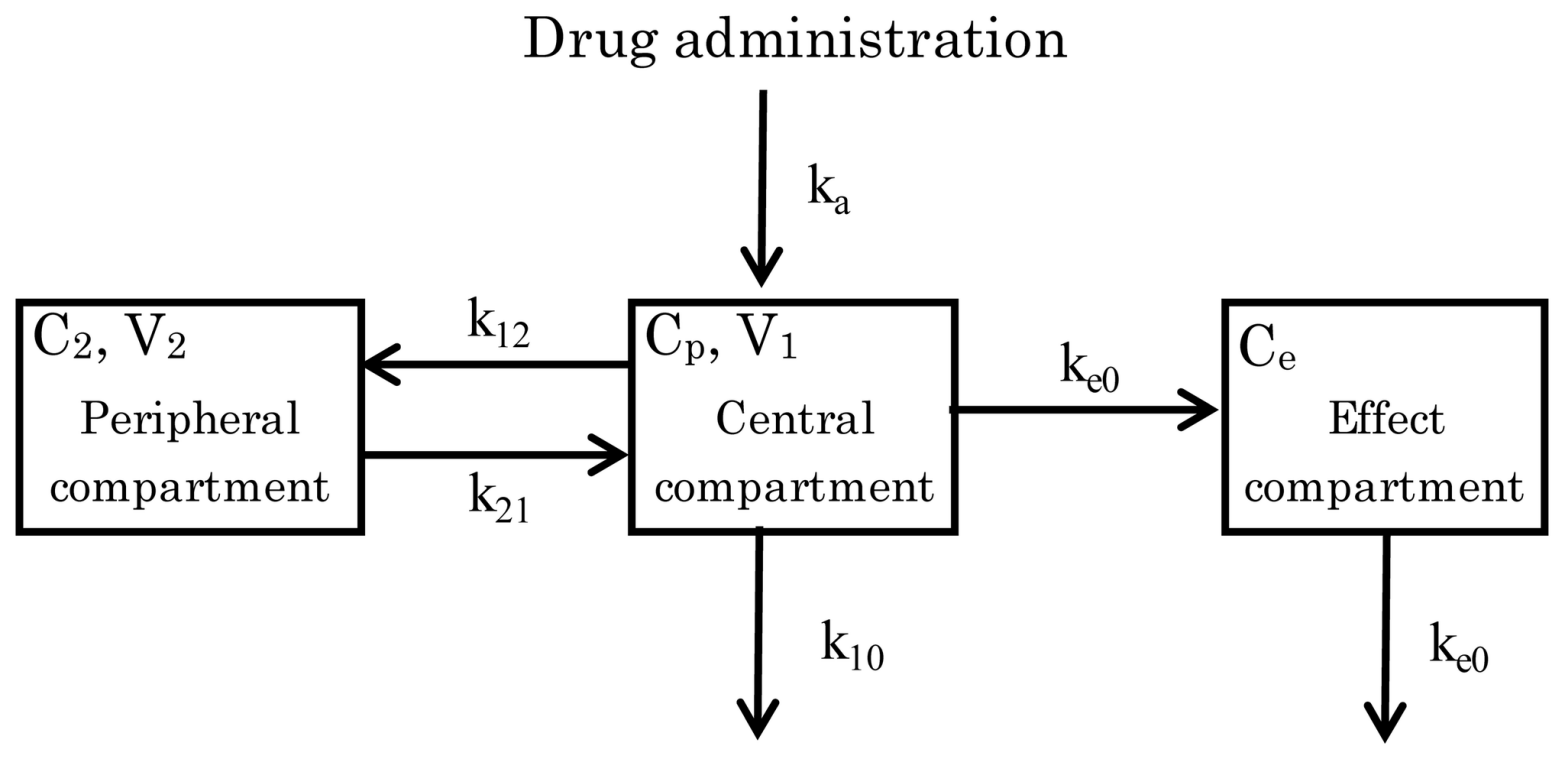
Schematic illustration of a model for description of E2110 PK/PD. C_p_, E2110 concentration in the central compartment; C_e_, E2110 concentration in the effect compartment; k_a_, absorption rate constant; k_e0_, equilibrium rate constant; V_1_, central volume of distribution; V_2_, peripheral volume of distribution.

The effect compartment model is illustrated in Figure 2. The mean plasma concentration profile of E2110 in each of four rats was fitted by two-compartment model with first order absorption and elimination. A sigmoid E_max_ model (Equation 4) based on the concentration in the effect compartment was fitted to the PD (RO) data. PK and PD models were linked using an effect compartment model [13] described by the following equation:

$$\frac{{dC}_{e}}{dt}=k_{e0}(C_{p}-C_{e})$$ (5)

where C_p_ and C_e_ are concentrations of E2110 in the plasma and effect compartments, respectively, and k_e0_ represents the equilibration rate constant for the effect compartment. PK and PD parameters were estimated by using SCIENTIST (Version 2.01, MicroMath Research, UT, USA).

### Pharmacological Tests

We used female SD rats for pharmacological assays to assess effects of E2110 on micturition functions, since insertion of a urinary catheter into the bladder could be readily performed in female rats.

#### 1) 5-HT_1A_ receptor agonist-induced model of OAB

Catheter implantations and cystometric investigations were performed based on the reported method [14]. Briefly, on the day before the cystometric experiment, female SD rats were initially anesthetized with pentobarbital (50 mg/kg ip). Catheters were inserted into the femoral vein, the dome of the stomach and the dome of the bladder. In the cystometric study, the bladder catheter was connected to an infusion pump and a pressure transducer (DX-312; Nihon Kohden Co., Tokyo, Japan) by three-way stopcock. Saline was infused into the bladder and femoral vein. After bladder pressure was stabilized, 8-OH-DPAT (0.06 mg/kg/h) was infused into the femoral vein instead of saline. Bladder pressure was monitored with a pressure transducer and a pressure amplifier (AP-601G; Nihon Kohden), and recorded on a pen recorder (WT-645G; Nihon Kohden). The following three micturition intervals (i.e., intervals between consecutive peaks of bladder pressure induced at the time of bladder content evacuation) were obtained: (1) basal micturition interval (intervals before 8-OH-DPAT administration), (2) pre-administration micturition interval, (intervals immediately before administration of E2110 or vehicle), and (3) post-administration micturition interval (intervals observed between 1 and 2 hr after administration of E2110 or vehicle). The change in the micturition interval after E2110 administration was used as an index of the efficacy of the treatment. E2110 (0.03, 0.1 or 0.3 mg/kg) or vehicle was administered to four groups of eight conscious rats orally.

#### 2) Superior colliculus lesion (SCL) model

For lesioning of the brain to stimulate the micturition reflex, anesthesia was induced in female SD rats with 4% halothane and an N_2_O/O_2_ (2:1) gas mixture, it was maintained with 2% halothane, and each animal was mounted onto a stereotaxic apparatus. The skull was then exposed, and holes were drilled for the superior collicular placement (anterior = + 2.0 mm; lateral = ±1.7 mm; horizontal = 0.0 mm) of a lesion electrode (TM-type tip; 0.7 mm × 1.5 mm). The bilateral superior colliculi were lesioned by electrical heating at 65°C for 4 min with a lesion generator (RFG-4; Muromachi Kikai Co., Ltd., Tokyo, Japan). Catheter implantations and cystometric investigations were performed with the same methodologies as in the 5-HT_1A_ receptor agonist-induced model. E2110 (0.1, 0.3 or 1 mg/kg) or vehicle was administered to four groups of eight conscious rats orally. The three micturition intervals were obtained and drug-induced changes in the micturition intervals were evaluated. In this assay, the post-administration micturition interval was defined as the average of the micturition intervals over an observation period between 0.5 and 1 hour after administration.

### Data Presentation and Statistical Analysis

Data are expressed as mean ± standard error of mean (S.E.M). In the pharmacological study, the statistical significance of differences between the vehicle- and E2110-treated groups was tested using one-way analysis of variance (ANOVA) followed by Dunnett’s multiple comparison test. A probability (p) value of <0.05 (two-sided) was considered statistically significant. All statistical analyses were performed using the SAS software package version 8.1 or 8.2 (SAS Institute Japan Ltd., Tokyo, Japan).

## Results

### Binding assay in rat hippocampal microsomal fraction

**Figure 3:**
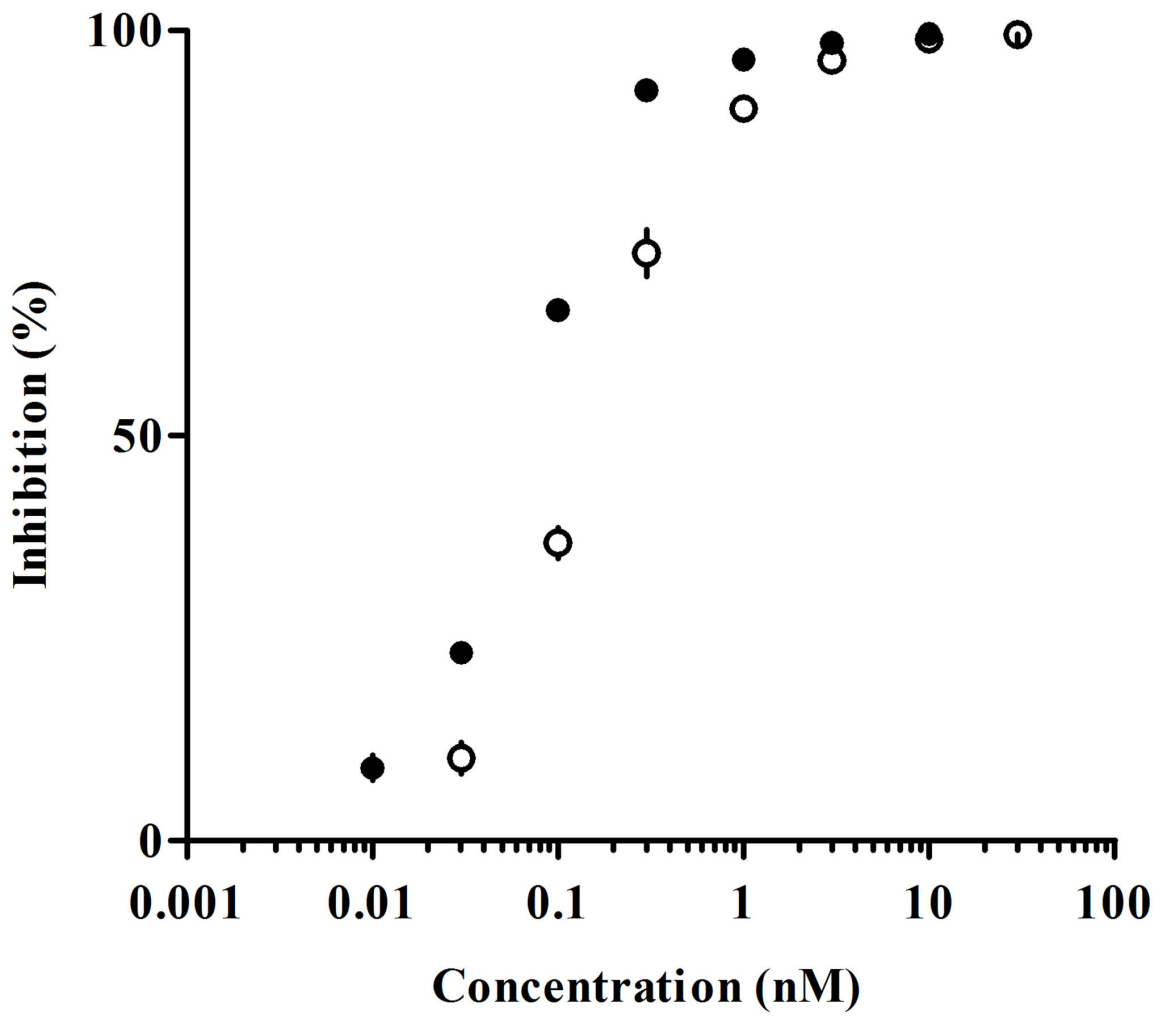
*In vitro* inhibition profiles of E2110 and WAY-100635 on 5-HT_1A_ receptors. Inhibition of specific [^3^H] MPPF binding in rat hippocampal membrane homogenates was measured at various concentrations of E2110 (●) and WAY-100635 (○).

The affinity of E2110 for rat 5-HT_1A_ receptors was examined in brain tissues. [^3^H] MPPF bound to membrane preparations from the rat hippocampus with a K_d_ value of 0.92 nM. E2110 and WAY-100635 inhibited this [^3^H] MPPF binding in a concentration-dependent manner (Figure 3). K_i_ values of E2110 and WAY-100635 for rat 5-HT_1A_ receptor were 0.045 nM (Table 1) and 0.121 nM, respectively.

**Table 1:** *In vitro* pharmacological profile and *in vivo* PD parameter estimates for E2110.

	*In vitro*				*In vivo*		
	5-HT_1A_ receptor affinity			ROI	5-HT_1A_ RO		
					Dose-response study		Time-course study
Ki (nM)	0.045		EC_50_ (nM)	MPFC	7.51 (0.45)		5.85 (0.35)
				DRN	5.40 (0.32)		7.38 (0.44)

### 5-HT_1A_ RO Studies

**Figure 4:**
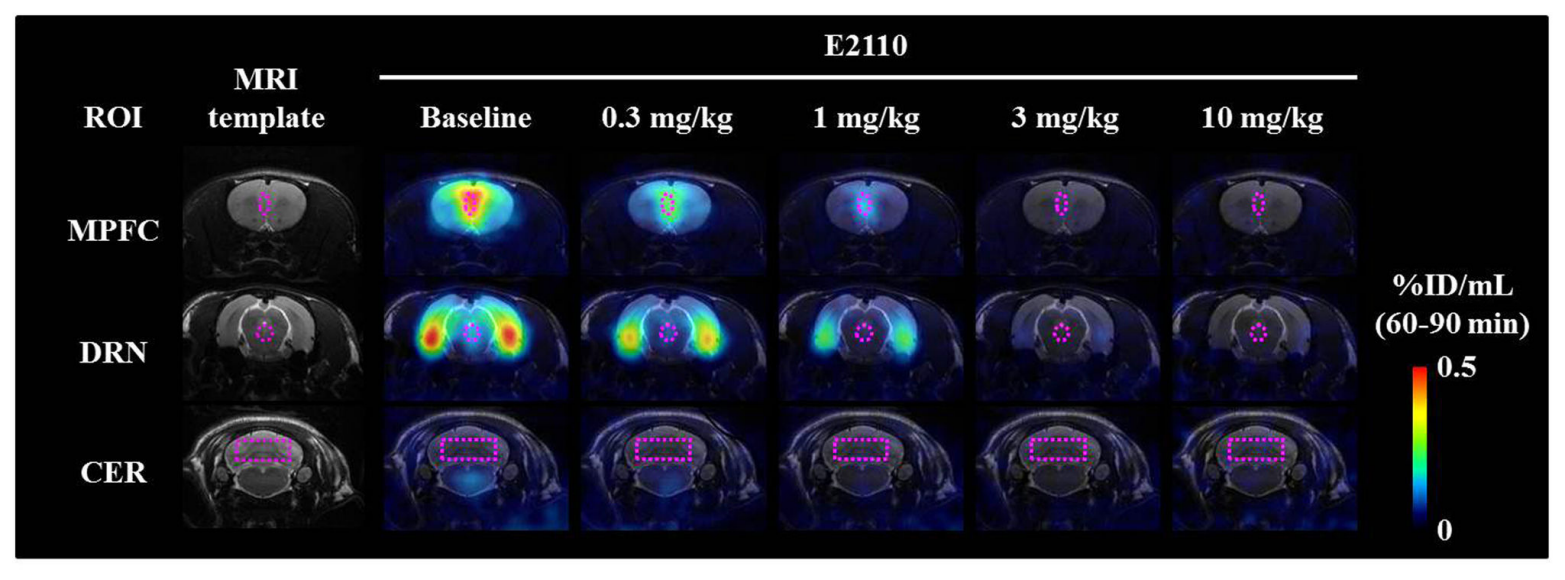
Representative PET images illustrating distribution of [^11^C] WAY-100635 in rat brains at baseline and after oral administration of E2110. PET images were generated by averaging dynamic data at 60-90 min after intravenous radiotracer injection, and were overlaid on the MRI template shown in the far left column. Coronal brain sections shown here were obtained at 1.0 mm (top row), -7.8 mm (middle row) and -12.5 mm (bottom row) from the bregma. ROIs (dotted lines) were defined on the MPFC (top row), DRN (middle row) and cerebellum (CER; bottom row). The radiotracer retention was presented as a percentage of the injected dose per unit tissue volume (%ID/mL).

**Figure 5:**
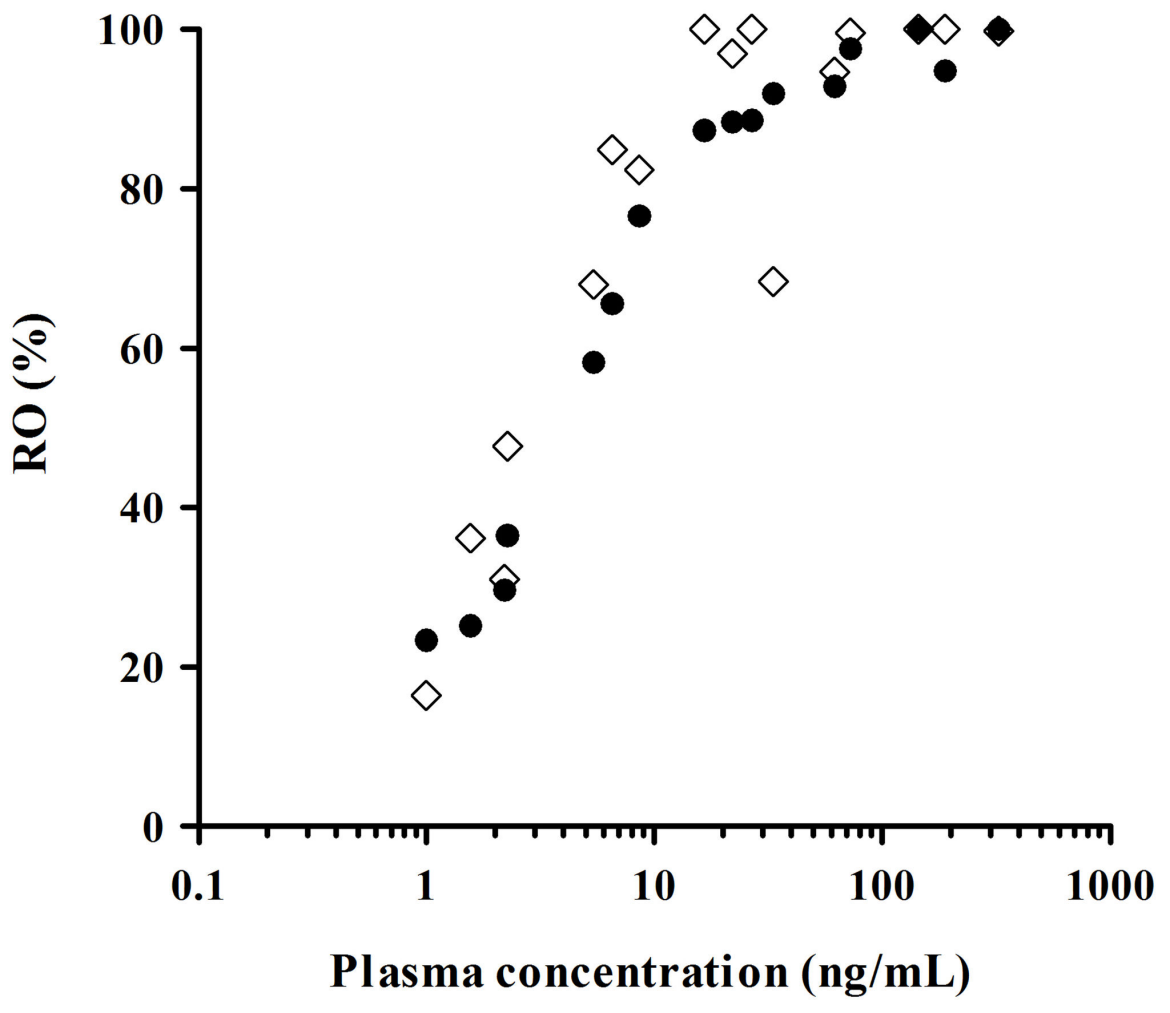
Relationship between rat 5-HT_1A_ RO (MPFC: ●, DRN: ◇) and E2110 plasma concentration. 5-HT_1A_ RO was determined at 4 hours after oral administration of E2110 at a dose ranging from 0.3 to 10 mg/kg. Symbols represent individual data from all dose levels (n = 4/dose level).

As MPFC and DRN represent brain areas enriched with post- and pre-synaptic 5-HT_1A_ receptors, respectively, ROIs were defined in these areas for subsequent analyses. As visually demonstrated by inhibition of [^11^C] WAY-100635 binding in representative PET images (Figure 4), E2110 dose-dependently induced 5-HT_1A_ RO at single oral doses of 0.3, 1, 3, and 10 mg/kg in each region. The relationship between the plasma concentration of E2110 and its occupancy of 5-HT_1A_ receptors could be described by hyperbolic function (Equation 4, Figure 5). Since high doses of E2110 (3 and 10 mg/kg) induced full occupancy of 5-HT_1A_ receptors in both ROIs, E_max_ (RO_max_) was fixed at 100%. By applying this E_max_ model, the E2110 plasma concentration required for 50% RO (EC_50_) was estimated to be 3.68 ng/mL (7.51 nM) and 2.64 ng/mL (5.40 nM) in MPFC and DRN, respectively (Table 1).

### Protein binding and PK in rat plasma

The binding of E2110 at 0.1, 1 µM to plasma proteins was not dependent on its concentration. The average percentage of protein-unbound E2110 in plasma was 6%.

**Figure 6:**
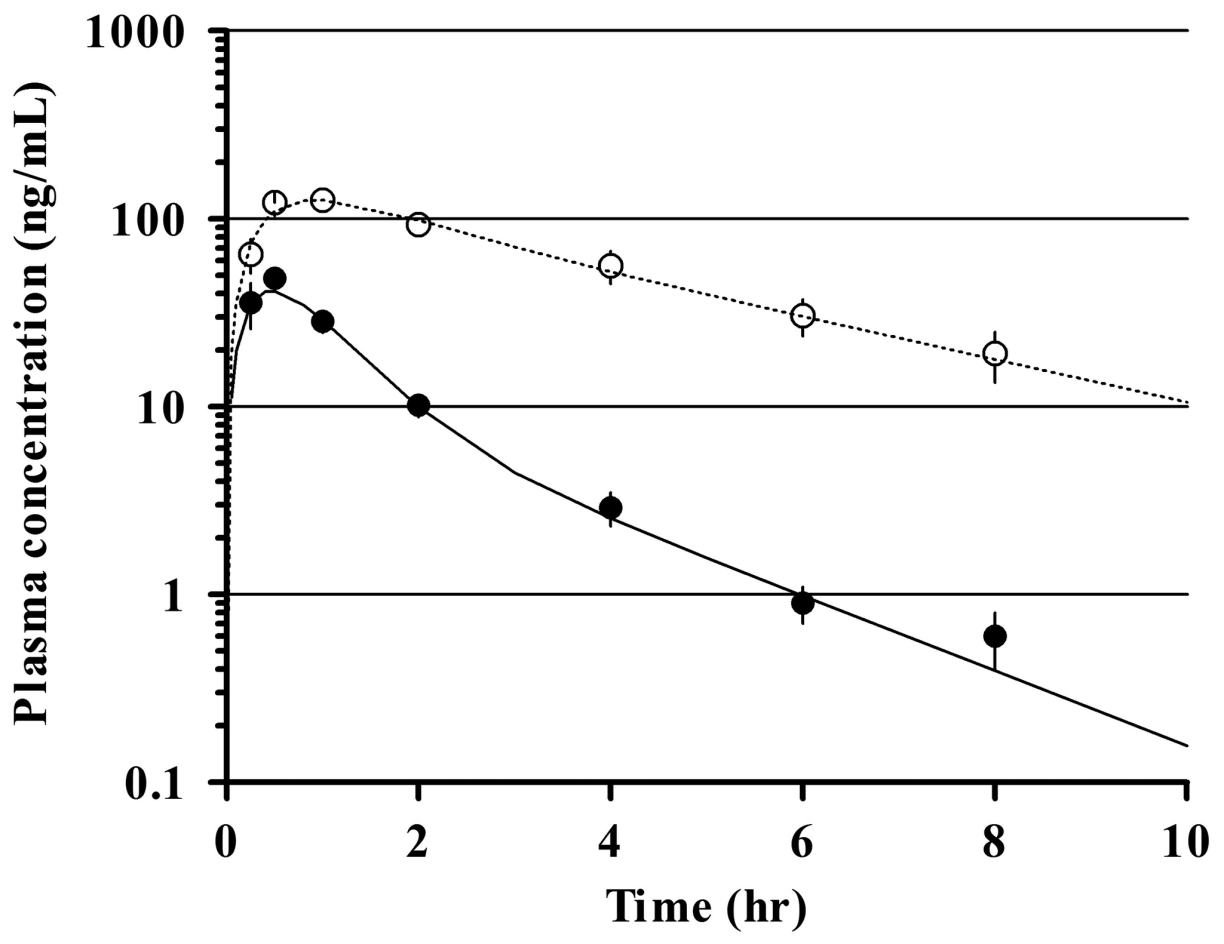
Plasma PK profile of E2110 after oral administration to rats. Symbols denote observed plasma concentrations (mean±S.E.M, n = 4/time point) in male (●) and female SD rats (○). Lines represent fitting of a two-compartment PK model with first-order absorption to the experimental data.

Male and female SD rats were given a single oral dose of E2110 (1 mg/kg). The time course of mean plasma concentrations was reasonably well described by a two-compartment model with first-order absorption, as displayed in Figure 6. E2110 was rapidly absorbed following oral administration, with peak concentrations in plasma (C_max_) occurring within 1 hour after dosing in all groups of rats. Elimination of the drug from plasma occurred more slowly in females than in males. The PK parameters for E2110 derived from these data, consisting of half-life in plasma (t_1/2_), maximum concentration (C_max_), time at maximum concentration (T_max_), and area under the curve in the time course of plasma drug concentration from 0 to 8 hours after oral administration (AUC_0-8hr_), are summarized in Table 2.

**Table 2:** PK parameter estimates after oral administration of E2110 to male SD, female SD rats and female SHRs.

Pharmacokinetic parameters		Male SD		Female SD	
t_1/2_ (hr)		1.5		2.6	
C_max_ (μg/mL)		48.1		125.7	
T_max_ (hr)		0.5		1	
AUC_0-8hr_ (μg•hr/mL)		71.8		487.9	

### PK/PD modeling and simulation

**Figure 7:**
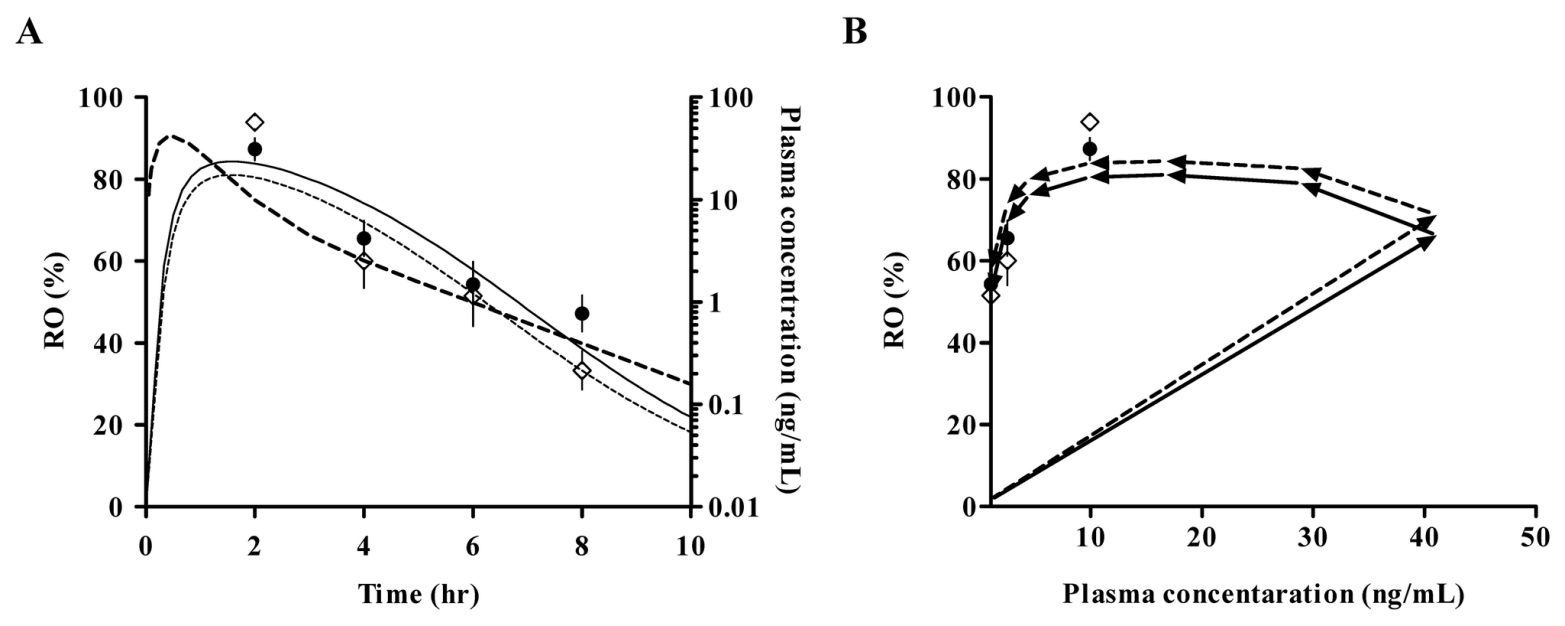
Time course data of rat 5-HT_1A_ RO in MPFC (●) and DRN (◇), and plasma E2110 concentration (thick dashed line) (A), and plot of RO against plasma E2110 concentration at individual time points (B). 5-HT_1A_ RO was determined at assigned time points after oral administration of E2110 at a dose of 1 mg/kg. Symbols represent mean ± S.E.M at indicated time points (n = 4/time point). Lines indicate the predicted occupancy versus plasma concentration in MPFC (solid line) and DRN (dashed line).

**Figure 8:**
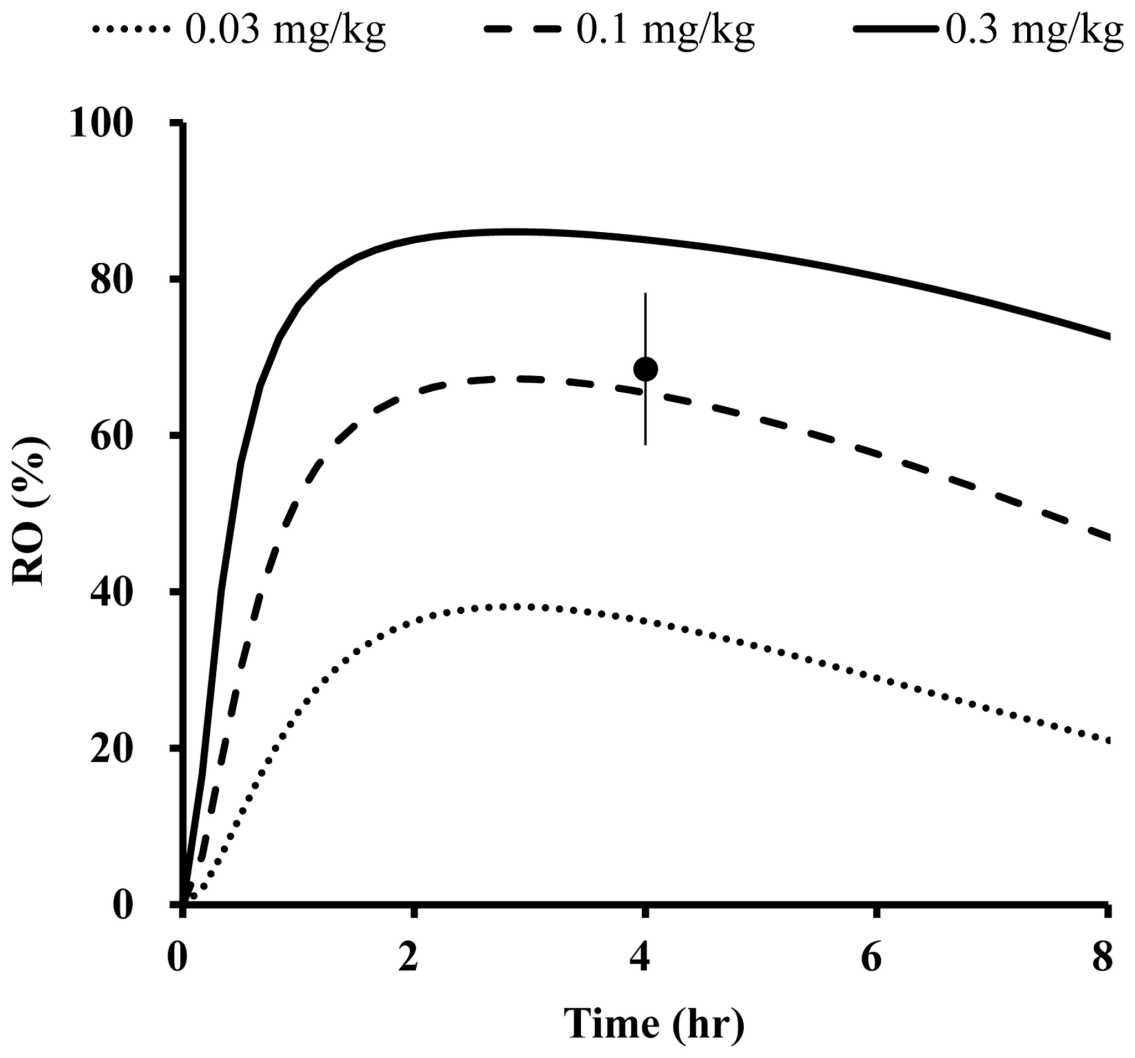
Effect compartment model estimation of 5-HT_1A_ RO in DRN after oral administration of E2110 at doses of 0.03, 0.1 and 0.3 mg/kg to female rats. Solid circle and error bars represent mean RO ± S.E.M measured by PET scans in female rats (n = 3).

In addition to the plasma concentration of E2110, its occupancy of 5-HT_1A_ receptors (Figure 7A) was monitored in four male SD rats over the time course of 8 hours after a single oral dosing (1 mg/kg). The two-compartment model with first-order absorption fitted to the temporal profile of the mean plasma concentration of E2110 was then linked to an effect compartment sigmoidal E_max_ model (Figure 2). The peaking and subsequent decline of 5-HT_1A_ RO by E2110 were somewhat retarded relative to the plasma kinetics (Figure 7A), and this slight delay was also indicated by the hysteresis curve (Figure 7B). Estimated PD parameters derived from this analysis are listed in Table 1. The EC_50_ value for occupancy of 5-HT_1A_ receptors by E2110 in MPFC and DRN were estimated to be 2.87 ng/mL (5.85 nM) and 3.6 ng/mL (7.38 nM), respectively. Despite the time lag between the plasma PK and brain RO, these values were rather close to those estimated by the direct model, indicating that there were no marked hysteresis effects. These PD parameters were utilized to simulate the 5-HT_1A_ RO versus time profiles in female SD rats (Figure 8). In order to assess the validity of this simulation data for predicting RO by E2110, we additionally performed a pilot PET study for female rats to determine 5-HT_1A_ RO at 4 hours after oral administration of 0.1 mg/kg of E2110, and found that RO values measured by PET were in good agreement with calculated data (Figure 8), justifying the use of simulation curves to estimate RO in female rats.

### Effects of E2110 on micturition reflex in rats

**Figure 9:**
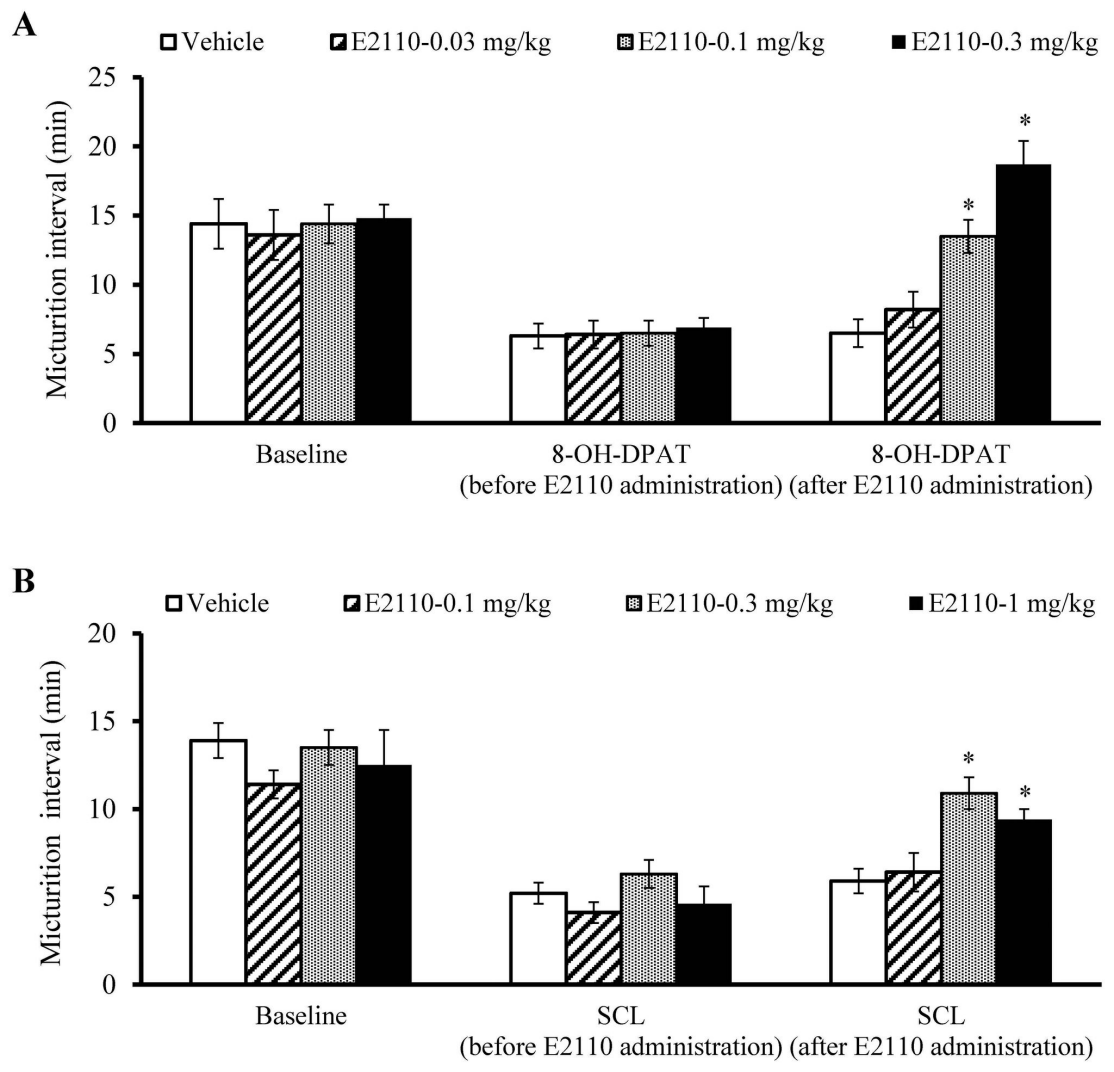
Effects of E2110 on micturition interval in 8-OH-DPAT-infused (A) and SCL (B) rats. Values are expressed as mean ± S.E.M. of eight rats in 8-OH-DPAT-infused and SCL models; * *P* < 0.05 versus vehicle (Dunnett’s multiple test).

The effects of E2110 on urinary function were evaluated in rats. The micturition intervals in female SD rats were markedly decreased from the basal level by intravenous infusion of 8-OH-DPAT (n = 8; Figure 9A) or SCL (n = 8; Figure 9B). Oral administration of E2110 at doses of 0.1-0.3 mg/kg in 8-OH-DPAT treatment and 0.3-1 mg/kg in the SCL model experiment significantly (p < 0.05) prolonged the micturition intervals. According to the simulation data (Figure 8), 0.1 mg/kg of E2110 induced 60-70% RO at 1-2 hours after dosing in 8-OH-DPAT treatment, and 0.3 mg/kg of E2110 resulted in 60-80% RO at 0.5-1 hour after dosing in SCL model. From this, it can be inferred that at least 60% RO is required for significant effects of E2110 on micturition.

## Discussion

In recent years, PK/PD modeling has been increasingly applied in drug discovery and early drug development. Preclinical PK/PD studies may prompt a series of important mechanistic studies to investigate the relationship between plasma concentrations and resulting effects on a biomarker in later stages of clinical drug development. The PK/PD modeling and simulation approaches to preclinical pharmacology studies may help to understand the mechanism of action of novel compounds and target engagement in preclinical models. The present data have demonstrated the utility and robustness of *in vivo* PET imaging techniques to monitor RO in living brains of small animals. Both direct and indirect models yielded a good fit to the experimental data and similar EC_50_ values for the drug under development, E2110, validating the use of these methodologies in rats and, potentially, humans. The dose-dependent effects of E2110 on bladder micturition function were consistently explained by the time course of RO induced by the drug, allowing prediction of pharmacologically efficacious doses of the drug by clinical PET estimation of RO.

Based on the results of our dose-response PET study conducted at a fixed time point after E2110 treatment, 5-HT_1A_ RO was well fitted by a simple E_max_ model, and the free concentration-based EC_50_ value did not markedly differ between MPFC (0.22 ng/mL, 0.45 nM) and DRN (0.16 ng/mL, 0.32 nM). These findings indicate that E2110 has equal antagonist activities against pre- and post-synaptic 5-HT_1A_ receptors. Meanwhile, these free concentration-based EC_50_ values were approximately 10-fold of the K_i_ value (0.045 nM) calculated from the *in vitro* binding assay for 5-HT_1A_ receptor. Our supplemental study indicated that E2110 is a substrate for the BBB drug transporter P-glycoprotein in rodents, as the brain-to-plasma concentration ratio (K_p, brain_) of E2110 concentration in Mdr1a/1b-knockout mice was ten times higher than that in wild-type mice (data not shown). Therefore, the free E2110 concentration in CNS tissues is assumed to be one-tenth of its free plasma concentration. On the basis of this assumption, the *in vivo* EC_50_ values corrected for the difference in free drug concentrations between plasma and CNS were estimated to be 0.045 and 0.032 nM in MPFC and DRN, respectively, and these values are comparable with *in vitro* K_i_. This finding also supports the reliability of PET assays for anesthetized rats to quantify 5-HT_1A_ RO. Indeed, the use of isoflurane-anesthetized rats for 5-HT1A RO determination was justified in a previous [^11^C] WAY-100635-PET study, which demonstrated that *in vivo* ED_50_ value for pindolol in the hippocampus (5.6 mg/kg) was rather close to hippocampal ED_50_ value (8.5 mg/kg) estimated by an *ex vivo* autoradiographic measurement for unanesthetized rats [15].

The time course of 5-HT_1A_ RO and plasma PK after a single oral dose of E2110 (1 mg/kg) offered details of the PK-RO relationships for this drug, and enabled simulation of RO in pharmacological models. This effect compartment model well described the temporal profiles of 5-HT_1A_ RO, and the EC_50_ values based on this PK/PD modeling were estimated to be 2.87 ng/mL (5.85 nM) and 3.6 ng/mL (7.38 nM) in MPFC and DRN, respectively. Hysteresis was observed in a plot of the relationship between plasma concentration and 5-HT_1A_ RO, and this lag time is typically observed for centrally acting compounds, due to either rate-limiting BBB passage of the compounds or slow receptor on/off targets [16]. Although such delay was observed between RO and plasma PK of E2110, the EC_50_ values calculated with the direct and effect compartment models were nearly equivalent. Additionally, estimates of the equilibration rate constant (k_e0_) were relatively large, indicating a rapid equilibration with the CNS biophase (CNS equilibration t_1/2_ of 1.4 hours). Therefore, hysteresis effects on the target binding of E2110 were minor in the present study.

The present work also demonstrated a profound difference in plasma PK profiles of E2110 between male and female SD rats (Table 2). Overt gender differences in expression levels of enzymes involved in drug metabolisms were reported in a previous rat study [17], and Chovan et al. documented that substrates of human drug-metabolizing enzyme cytochrome P450 3A4 (CYP3A4) were mainly metabolized by cytochrome P450 3A1/2 and 2C11 in rats [18], and expression levels of these enzymes in male rats are known to be higher than in female rats [17]. In addition, our unpublished results indicated that E2110 was primarily metabolized by CYP3A4 in human liver microsomes. Taking these findings together, we conceive that the difference in plasma PK of E2110 between male and female rats is attributable to gender difference in the levels of hepatic cytochromes responsible for metabolism of this drug. Despite these effects of gender on PK, 5-HT_1A_ RO in the female rat brain measured by PET was close to a value calculated by effect compartment model with k_e0_ determined in male rats (Figure 8), suggesting consistency of the PK-RO relationships between males and females.

Our findings in pharmacological tests have provided further support to the implication of 5-HT_1A_ receptors in the control of micturition in rats [19,20]. Numerous rat studies have indicated that 5-HT_1A_ receptors have an excitatory physiological role in modulating micturition [21]. Both spinal and supraspinal 5-HT_1A_ receptors constitute an efficient way to stimulate rat micturition. However, the modulation of the 5-HT_1A_ autoreceptor has been proposed to interfere with the micturition reflex [19,22]. Administration of 8-OH-DPAT was reported to facilitate the voiding reflex, and this effect was inhibited by the pretreatment with 5-HT_1A_ receptor antagonists such as WAY-100635 and NAD-299 [14,23]. The inhibitory effect of E2110 on the micturition reflex observed in the present work could accordingly be attributed to its action on supraspinal 5-HT_1A_ autoreceptors.

In order to correlate the degree of 5-HT_1A_ RO by E2110 yielding significant effects in pharmacological tests, we applied simulated temporal profiles of RO in DRN enriched with putative autoreceptors after oral doses of E2110 in female SD rats (0.03, 0.1, 3 mg/kg). The minimum doses of E2110 exerting overt therapeutic effects on the micturition reflex in 8-OH-DPAT and SCL models were 0.1 and 0.3 mg/kg, respectively. In reference to the simulated RO values, RO at these doses were estimated to be around 60% during the micturition monitoring period (at 1-2 hours postdose in the 8-OH-DPAT model and 0.5-1 hour postdose in the SCL model), demonstrating that RO around 60% or more was necessary for a significant effect on bladder function. Thus, for humans, the dosage of a 5-HT_1A_ antagonist would need to be determined to achieve this range of RO as a requirement for therapeutic efficacies against OAB.

In summary, our data support that occupancy of central 5-HT_1A_ receptors quantified by *in vivo* PET is a useful biomarker surrogating anti-OAB effects of 5-HT_1A_ receptor antagonists. Although micturition is supposedly regulated by diverse neurotransmitter pathways at different levels in the central and peripheral nervous systems, the assessment PK-RO relationship using brain PET data would help to determine and/or predict effective doses of these candidate drugs in rat models. Meanwhile, several factors, including species differences in the protein-unbound fraction and the presence or absence of an active metabolite of such a provisional drug, should be taken into account in consideration of homology between EC_50_ values in laboratory animals and humans. Species differences in RO and PK-RO relationship would be assessable with the aid of comparative PET assays.
